# Construction of a scoring system for predicting the risk of severe gastrointestinal involvement in Henoch-Schönlein Purpura

**DOI:** 10.1186/2193-1801-3-171

**Published:** 2014-04-02

**Authors:** Tsunehisa Nagamori, Hideharu Oka, Shin Koyano, Hironori Takahashi, Junichi Oki, Yuko Sato, Koichi Murono, Kenichi Iseki, Ryou Takeguchi, Takahiro Takeda, Masayuki Sato, Rika Sugai, Hiroyuki Kitamura, Hiroki Kajino, Yurika Miura, Toru Ishioka, Hiroshi Azuma

**Affiliations:** Department of Pediatrics, Asahikawa Medical University, Asahikawa, Hokkaido, Japan; Department of Pediatrics, Asahikawa Kosei Hospital, Asahikawa, Hokkaido, Japan; Department of Pediatrics, Nayoro City Hospital, Nayoro, Hokkaido, Japan; Department of Pediatrics, Fukagawa City Hospital, Fukagawa, Hokkaido, Japan; Department of Pediatrics, Wakkanai City Hospital, Wakkanai, Hokkaidō, Japan; Department of Pediatrics, Extended Mombetsu City Hospital, Mombetsu, Hokkaido, Japan; Department of Pediatrics, Abashiri Kosei Hospital, Abashiri, Hokkaido, Japan; Department of Pediatrics, Engaru Kosei Hospital, Hokkaido, Japan

## Abstract

**Objective:**

To evaluate the parameters associated with significant gastrointestinal (GI) involvement in Henoch-Schönlein Purpura (HSP), and construct a scoring system for the identification of patients at high risk of gross blood in stools.

**Study design:**

Data for HSP patients hospitalized at each of seven institutes were retrospectively analyzed. Patients were divided into four groups according to the consequent severity of GI involvement. Identification of laboratory parameters at the time of admission were then used to differentiate the groups, and a scoring system to predict gross intestinal bleeding was constructed. Prognostic efficiency, correlation with the subsequent duration of abdominal pain, and association with manifestations excluding abdominal pain were also analyzed.

**Results:**

An analysis of variance (ANOVA) test showed significant intergroup differences in white blood cell (WBC) count, neutrophil count, serum albumin, potassium, plasma D-dimer and coagulation factor XIII activity. A scoring system consisting of these parameters showed a good prognostic value for gross intestinal bleeding in a receiver operating characteristic (ROC) analysis, and a cut-off value of 4 points showed a sensitivity of 90.0% and specificity of 80.6%. The score was also correlated with the duration of abdominal pain after admission. A significantly higher score (s) was observed in patients presenting with nephritis, although the predictive value was poor.

**Conclusion:**

A scoring system consisting of generally available parameters was of use in predicting severe GI involvement in HSP patients. Although further study is needed, initial therapy in accordance with disease activity may be taken into consideration using this scoring system.

## Introduction

Henoch-Schönlein Purpura (HSP) is a systemic, IgA-mediated, small vessel vasculitis which is common in children (Saulsbury 2001). Clinical manifestations including non-thrombocytopenic purpura, gastrointestinal (GI) involvement and arthralgia are common in the early phase of HSP. Further, HSP nephritis (HSPN), the onset of which may be delayed for weeks or months after the appearance of other symptoms, is the most serious long-term complication of HSP (Narchi 2005). Although HSP generally follows a benign, self-limiting course that is resolved within a few weeks, some cases present with severe GI involvement including massive intestinal bleeding that can lead to acute complications such as intestinal perforation or obstruction (Choong & Beasley 1998; Saulsbury 2007). Severe GI involvement presenting as gross blood in stools in the early phase of HSP is important, not only because of the distress it causes patients, but also because it is a significant risk factor for the subsequent onset of HSPN (Kaku et al. 1998; Sano et al. 2002; Bogdanovic 2009). However, the assessment of GI involvement can be often difficult due to the nature of the abdominal pain. It is colicky and often fluctuates, and the common presentation of blood in stools can be delayed for several days after the onset of other symptoms (Zhang & Huang 2008).

Previous reports have already demonstrated that HSP patients present with a high serum IgA concentration (Saulsbury 1999; Calvino et al. 2001; Trapani et al. 2005), low C3 or C4 (Calvino et al. 2001; Trapani et al. 2005), and leukocytosis (Trapani et al. 2005). Furthermore, a decrease in fXIII activity (Kamitsuji et al. 1987), and increases in prothrombin fragment 1 + 2 and D-dimer levels (Yilmaz et al. 2005) were shown to be associated with the severity of GI involvement. Unfortunately, however, the sole predictive value of these parameters for intestinal bleeding has not yet been demonstrated.

In the present study, we conducted a multi-centered retrospective analysis of 140 patients with HSP to evaluate the parameters associated with significant GI involvement, and construct a scoring system for the identification of patients at high risk for such involvement.

### Patients and methods

#### Study population

We retrospectively reviewed the medical records of HSP patients hospitalized between January 2003 and December 2012 at seven institutions in Hokkaido, Japan. A diagnosis of HSP was made on the basis of the presence of the major manifestations of the illness, consisting of purpura and abdominal pain or arthralgia without thrombocytopenia (Saulsbury 1999). HSPN was defined by the presence of gross or microscopic hematuria (>5 cells per high power field from a centrifuged specimen) either with or without proteinuria (Saulsbury 1993). Patients were excluded on the basis of severe complications affecting abdominal pain or a lack of laboratory data. Patients representing with abdominal pain later than two days after hospitalization were also excluded, in consideration of their laboratory data not reflecting actual disease activity. Fecal hemoglobin was repetitively examined as long as abdominal pain continued. The duration of abdominal pain after admission was defined as the number of hospitalization day(s) the patient suffered from abdominal pain without remission for a 24-hour interval.

#### Therapeutic strategy for early phase HSP

The essential therapeutic strategy for early phase HSP on admission was basically concordant among the participating institutes in that patients were recommended bed rest, and oral or intravenous prednisolone (PSL) 1.5 ~ 2 mg/kg/day was administrated when the patient complained of unacceptable abdominal or joint pain.

#### Four patient groups categorized according to gastrointestinal involvement

Patients were divided into four groups based on the severity of gastrointestinal (GI) involvement as follows; Group I: no abdominal pain, Group II: presence of abdominal pain, without intestinal bleeding (negative for fecal hemoglobin), Group III: abdominal pain and positive for fecal hemoglobin, but not presenting with gross blood in the stools, and Group IV: abdominal pain with gross blood in the stools.

#### Data analysis

All analyses were carried out using the SPSS statistical software package, version 16.0 (SPSS, Chicago, IL, USA). Data are presented as the mean ± standard deviation (SD) or median and range for continuous variables, or as the percentage of patients showing a given categorical variable. For all analyses, a 2-sided probability value below 0.05 was considered to indicate statistical significance. Spearman’s rank correlation coefficient test was used to examine correlations among categorical parameters among the baseline characteristics and GI-involvement groups. A Kruskal-Wallis analysis of variance (ANOVA) was used to examine overall differences in laboratory data at the time of admission and other continuous variables among the GI-involvement groups. The data included WBC count, neutrophil count, hemoglobin, platelet count, serum concentration of albumin, aspartate aminotransferase (AST), alanine aminotransferase (ALT), lactate dehydrogenase (LDH), and C-reactive protein (CRP), prothrombin time (PT), activated partial thromboplastin time (APTT), plasma concentration of fibrinogen and D-dimer, coagulation factor XIII (fXIII) activity, serum concentration of antistreptolysin O antibody (ASO), IgA, complement C3, C4, total hemolytic complement (Ch50), sodium, and potassium. And age, days from symptom onset to admission, and days from abdominal pain onset to admission was also extracted. We then constructed a scoring model with which to identify severe GI involvement consisting of the parameters found to be statistically significant in the ANOVA analysis. A receiver operating characteristic (ROC) analysis was also carried out to examine the diagnostic utility of the constructed scoring model, and the Mann–Whitney *U* test was used to compare scores between patients with and without arthralgia and nephritis. The correlation between duration of abdominal pain after admission and score at admission was also examined using Spearman’s rank correlation coefficient.

## Results

### Patient characteristics

During the study period, 140 patients were analysed, with 27 later excluded (due to complications of intussusception in 3, acute pancreatitis in 1, testicular torsion in 1, lack of data in 19, and presentation of abdominal pain later than two days after admission in 3 patients). Thus, 113 patients were included in the study. Table 1 shows the baseline characteristics of the patients by GI-involvement group. Although a significant correlation was observed between the development of HSPN and gender, with males more likely to develop HSPN, no significant differences were found among GI-involvement groups for any of the patient characteristics.

**Table 1:** **Baseline characteristics and gastrointestinal involvement (GI) groups**

Baseline characteristics of the patients				
N	113			
Age, median	6 (1-15)			
(Years) (range)				
SEX (M/F)	51/62			
Duration from symptom onset to admission (days) (mean ± SD)	5.8 ± 5.2			
Purpura (%)	113 (100%)			
Arthralgia (%)	49 (43%)			
Abdominal pain (%)	54 (47%)			
GAS infection (%)	18 (15%)			
HSPN (%)	20 (18%)			
**Gastrointestinal Involvement Groups**				
	Group I	Group II	Group II	Group IV
N	57	24	21	11
Age, medican	6 (1-15)	5.5 (2-13)	7 (2.-10)	6 (2-11)
(Years) (range)				
Duration from symptom onset to admission	5.8 ± 5.6	5.4 ± 4.4	5.5 ± 5.5	7.5 ± 4.7
(Days) (mean ± SD)				
Purpura (%)	55 (100%)	23 (100%)	20 (100%)	10 (100%)
Arthralgia (%)	26 (46%)	7 (29%)	11 (52%)	4 (36%)
Abdominal pain (%)	-	23 (100%)	20 (100%)	10 (100%)
GAS infection (%)	11 (19%)	3 (13%)	4 (19%)	0 (0%)
PSL administration (%)	9 (15%)	15 (63%)	17 (81%)	10 (90%)
HSPN (%)	5 (9%)	8 (33%)	4 (19%)	3 (27%)
Significant correlation: HSPN vs Male	(R = 0.35, p = <0.01)			

GI groups were categorized as follows; Group I: no abdominal pain, Group II: abdominal pain without fecal hemoglobin, Group III: abdominal pain and positive for fecal hemoglobin, but negative for gross blood in stools, and Group IV: presence of gross blood in stools.

### Construction of a scoring system for the diagnosis of severe gastrointestinal involvement

**Figure 1:**
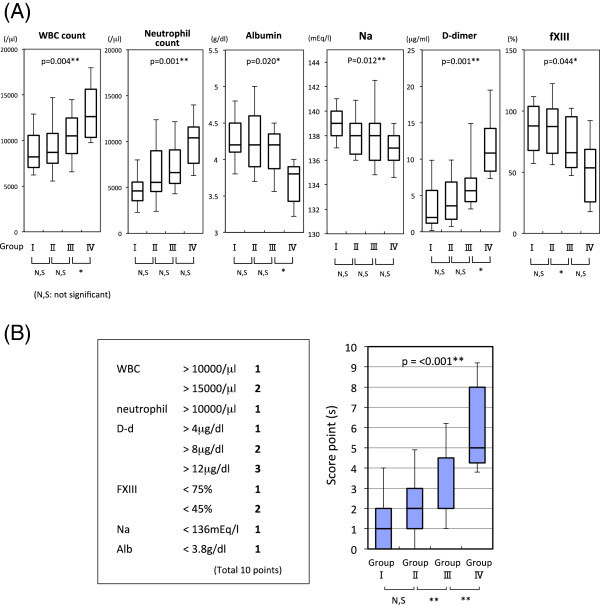
**Parameters showed overall differences among GI groups and construction of a scoring system. (A)** WBC count, neutrophil count, serum albumin, potassium, plasma D-dimer and coagulation factor XIII levels in each GI group are shown. These parameters showed overall significant differences by an analysis of variance (ANOVA) test. The probability values in the graph are for the ANOVA test, and those below the graph are for the Mann–Whitney *U* test between consecutive groups. Bars in the graph represents the 10th, 25th, median, 75th, and 95th percentiles. **(B)** The scoring system consisting of the above parameters (left) and scores at the time of admission for each GI group (right) are shown. The probability value in the graph is for the ANOVA test, and that below the graph are for the Mann–Whitney *U* test between consecutive GI groups. Bars in the graph represents the 10th, 25th, median, 75th, and 95th percentiles.

**Figure 2:**
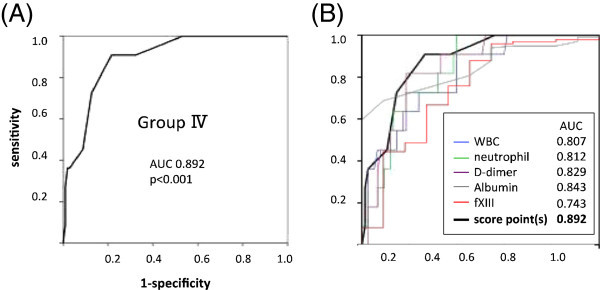
**Prognostic accuracy of scoring system for gross blood in stool. (A)** Receiver operating characteristic (ROC) curves of the scores for patients with gross blood in stools. **(B)** ROC curves and the areas under the ROC curves (AUCs) for each parameter comprising the scoring system are shown. The longitudinal axis shows the sensitivity for WBC count, neutrophil count, D-dimer and 1-specificity for albumin and coagulation factor XIII.

An ANOVA test showed the presence of significant overall differences in WBC count, neutrophil count, serum concentration of albumin, potassium, plasma level of D-dimer, and fXIII activity among the GI groups (Figure 1A). Construction of a scoring model consisting of these parameters was then carried out to increase prognostic utility (Figure 1B). The mean ± SD of each parameter and score(s) are shown in Table 2. Subsequently, ROC curves for Group IV were also evaluated (Figure 2). The area under the ROC curve (AUC) of score(s) for Group IV patients demonstrated good prognostic accuracy (AUC = 0.892). The construction of a scoring model improved diagnostic accuracy in comparison to that of each sole parameter in terms of AUC. To identify Group IV patients, the cut-off value was set at 4 points, a value which showed 90.0% sensitivity and 78.4% specificity.

**Table 2:** **The average and standard deviation of each parameter and the scores for the GI groups are shown**

mean ± SD	Group I	Group II	Group III	Group IV	P value
WBC count (/μl)	8938 ± 3057	9575 ± 3821	10575 ± 2993	13808 ± 4634	0.004
neutrophil count (/μl)	5068 ± 2626	6716 ± 3661	7324 ± 3147	10450 ± 3879	0.001
Albumin (g/dl)	4.22 ± 0.3	4.19 ± 0.5	4.06 ± 0.4	3.64 ± 0.5	0.020
D-dimer (μg/dl)	3.72 ± 3.6	6.22 ± 9.1	8.75 ± 9.0	11.75 ± 6.56	0.012
Coagulation factor XIII (%)	87.34 ± 26.5	87.80 ± 24.3	72.12 ± 25.2	57.48 ± 34.7	0.001
Sodium (mEq)l	138.86 ± 2.1	138.39 ± 2.0	137.36 ± 2.1	136.5 ± 2.3	0.044
**Score Point(s)**	**1.47 ± 1.5**	**2.17 ± 1.8**	**3.20 ± 2.1**	**5.8 ± 2.4**	**<0.001**

### Association between initial score and subsequent duration of abdominal pain

**Figure 3:**
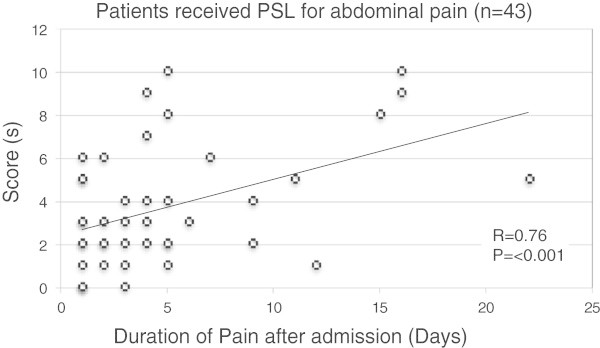
**Association between initial score and subsequent duration of abdominal pain.** Scatter plots of scores at the time of admission (longitudinal axis) and the subsequent duration of abdominal pain (horizontal axis) in 43 patients who received PSL therapy due to abdominal pain are shown.

In the study population, 56 patients (49%) presented with abdominal pain, placing them into Group II ~ IV, and 43 of them (76%) received PSL at 1.5 ~ 2 mg/kg/day. The scores at the time of admission for the 43 patients who received PSL for abdominal pain were significantly correlated with the subsequent duration of abdominal pain after admission (Figure 3).

### Association between initial score and subsequent development of HSPN

**Figure 4:**
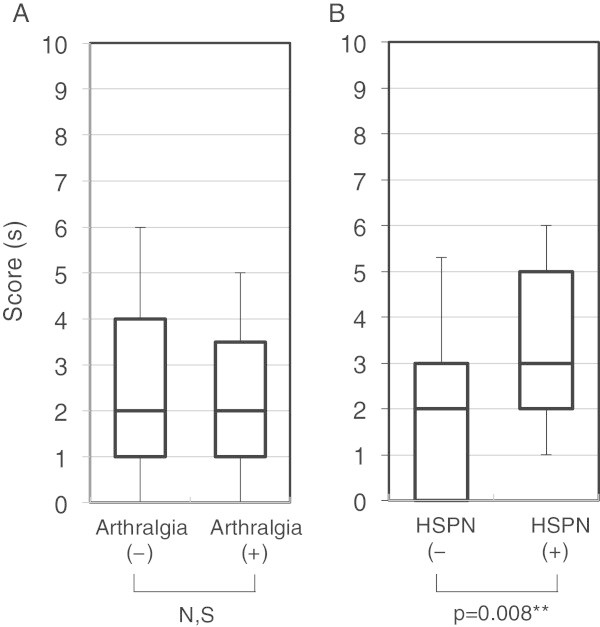
**Comparison of scores by HSP manifestations except for abdominal pain.** Scores compared between patients with and without **(A)** arthralgia (mean ± SD; 1.97 ± 1.94 and 2.38 ± 2.44, respectively) or **(B)** nephritis (average ± SD; 3.31 ± 1.89 and 2.15 ± 2.25, respectively).

A comparison of scores in association with manifestations of HSP excluding abdominal pain was also undertaken (Figure 4). There was no difference in scores between patients with or without arthralgia. A higher score was observed in patients with subsequent presentation of HSPN. However, the AUC for HSPN score showed only poor prognostic accuracy (AUC = 0.67, the ROC curve is not shown).

## Discussion

We focused on the prediction of significant GI involvement in the early phase of HSP, and found that our scoring system, consisting of generally available laboratory parameters, may be of value. A score of 4 points or higher, at the time of admission showed a good predictive value for gross blood in stools, and was associated with a longer duration of abdominal pain under PSL treatment. Based on its considerable predictive value for severe GI involvement, our scoring system has the potential to determine whether PSL intervention or supportive therapy, such as temporal fasting, should be undertaken. Further, as the monitoring of scores by repeat evaluation in several patients showed sequential changes parallel with GI symptoms (data not shown), it may also be useful for clinical follow up.

The precise mechanism underlying the changes in the parameters comprising the scoring system has not yet been clarified. Kawasaki disease, another systemic vasculitis common in children, often presents as hyponatremia and hypoalbuminemia, especially in severe cases (Fukunishi et al. 2000; Kobayashi et al. 2006). Further, patients with ulcerative colitis present with elevated D-dimer levels (Alkim et al. 2011; Zezos et al. 2009) and decreased fXIII activity (Lorenz et al. 1991), reflecting the inflammation of and injury to the intestinal mucosa. In consideration of these facts, our results might be regarded as reflecting severe vasculitis and the subsequent destruction of the small vessels, resulting in massive injury to the intestinal mucosa.

The efficacy of glucocorticoid therapy against the early symptoms of HSP remains controversial to a point. Several randomized controlled trials have reported that short-term PSL therapy (1.5 ~ 2 mg/kg/day) reduces the intensity of abdominal pain and arthralgia in early HSP (Ronkainen et al. 2006; Chartapisak et al. 2009; Rosenblum & Winter 1987). However, there have been several patient reports describing severe, persistent GI involvement despite appropriate PSL therapy (Saulsbury 1999; Taylor et al. 1971). As the proportion of patients with severe GI involvement is small, their refractoriness might be hidden behind the majority of benign cases. However, in terms of preventing HSPN, early identification and management of this small proportion of patients with severe GI involvement might be more important as, along with persistent purpura (Kaku et al. 1998; Sano et al. 2002; Rigante et al. 2005; Shin & Lee 2006; de Almeida et al. 2007), older age (Kaku et al. 1998; Sano et al. 2002; de Almeida et al. 2007), and decreased fXIII activity (Kaku et al. 1998; Sano et al. 2002), it is known to be an independent risk factor for HSPN (Kaku et al. 1998; Sano et al. 2002; Ronkainen et al. 2006; Shin & Lee 2006). As current PSL therapy in early HSP does not prevent subsequent HSPN (Ronkainen et al. 2006; Chartapisak et al. 2009; Rosenblum & Winter 1987; Huber et al. 2004), we propose the future evaluation of novel therapeutic strategies according to disease activity, and our scoring system might make an important contribution to this evaluation.

Of course, our study has several limitations. Our definition of GI group classifications was dependent on subjective symptoms and fecal examination; therefore, the classification criteria might require some improvement in terms of accuracy. Further, the proportion of patients with gross blood in stools was small. Last, our study was based on only a single cohort analysis. The survey of another cohort of HSP of HSP patients using our scoring system should help prove its validity. In addition, further large-scale or prospective studies are needed to overcome these limitations.

## Conclusion

The current study is the first report to the development of a scoring system for the identification of patients at risk for severe GI involvement on the basis of laboratory tests in the early phase of HSP.
